# Time course of changes in vision-related quality of life following intravitreal ranibizumab treatment for branch retinal vein occlusion

**DOI:** 10.1038/s41598-022-17587-0

**Published:** 2022-08-04

**Authors:** Shohei Morikawa, Fumiki Okamoto, Tomoya Murakami, Yoshimi Sugiura, Takahiro Hiraoka, Yoshifumi Okamoto, Tetsuro Oshika

**Affiliations:** 1 Department of Ophthalmology, Faculty of Medicine, University of Tsukuba, Tsukuba, Japan; 2 Department of Ophthalmology, Mito Kyodo General Hospital, Mito, Japan

**Keywords:** Retinal diseases, Vision disorders

## Abstract

To evaluate the vision-related quality of life (VR-QOL) treated by intravitreal ranibizumab (IVR) in patients with branch retinal vein occlusion (BRVO) and to assess subscale items of the VR-QOL. This was prospective, multicenter, open-label, observational study including 38 patients with unilateral BRVO who underwent IVR treatment and 28 age-matched healthy subjects. VR-QOL using the 25-item National Eye Institute Visual Function Questionnaire (VFQ-25) and best-corrected visual acuity (BCVA) were examined before and at 3, 6, and 12 months after treatment. The VFQ-25 composite score and BCVA significantly improved from 3 to 12 months after IVR treatment (*P* < 0.05), such that there was no significant difference between the BRVO and control groups at 12 months. All subscales of the VFQ-25, except “general health”, significantly improved after treatment, while “near vision” and “mental health” were worse than those in healthy subjects (*P* < 0.05). Patients with superior BRVO had a lower “near vision” score than healthy subjects after treatment (*P* < 0.05). BCVA in the treated eye and fellow eye had no significant relationship with the VFQ-25 composite score before and after treatment. The VR-QOL of patients with BRVO improved with IVR treatment and was comparable to that of healthy subjects after 12 months. Superior BRVO particularly affected near vision for a low level.

## Introduction

Branch retinal vein occlusion (BRVO) is a common retinal circulatory disease. Impairment of visual functions in patients with BRVO includes visual acuity impairment, metamorphopsia, contrast sensitivity, and stereopsis^1–4^. Even in cases where visual acuity is improved by treatment, BRVO could affect the quality of life (QOL) of patients by influencing factors such as other visual functions, treatment costs, and number of hospital visits.

The 25-item National Eye Institute Visual Function Questionnaire (VFQ-25) is a vision-specific health-related QOL instrument designed to assess patients’ perception of their vision-related function and the influence of vision problems on the performance of daily activities^5^. Suzukamo et al.^6^ developed a Japanese version of the VFQ-25 and proposed a particular combination of subscales based on the Japanese version’s factor structure and other psychometric characteristics. The VFQ-25 is composed of 12 vision-targeted health-related QOL domains and a VFQ-25 composite score is calculated. Prior studies have reported on the vision-related QOL (VR-QOL) in various retinal diseases such as epiretinal membrane (ERM)^7^, macular hole (MH)^8^, diabetic macular edema (DME)^9^, retinal detachment (RD)^10^, proliferative diabetic retinopathy (PDR)^11^, age-related macular degeneration^12^, and branch and central retinal vein occlusion^12–17^. The VFQ-25 composite score in patients with BRVO improved at 6 and 12 months after treatment by intravitreal ranibizumab (IVR) in the BRAVO study^13–15^ and at 52 weeks after intravitreal aflibercept in the VIBRANT study^16^. Awdeh et al.^17^ reported that all subscales of VFQ-25, except for “color vision”, were significantly worse in patients with BRVO than in healthy subjects before treatment. Varma et al.^15^ revealed that the subscale scores improved by an average of 10 points or more in “driving”, “general vision”, “social function”, “role difficulty”, “peripheral vision”, and “color vision” in patients with BRVO treated by IVR after 6 months.

However, the VR-QOL in patients with BRVO is poorly documented, and few clinical studies have revealed the subscales of VR-QOL in BRVO over a long-term period. In addition, to the best of our knowledge, there have been no reports comparing patients with BRVO and healthy subjects for VR-QOL after treatment. Therefore, the goal of the present study was to investigate the time course of change in VR-QOL over 12 months, the relationship between VR-QOL and visual acuity, and to evaluate the subscales of the VFQ-25 in patients with BRVO.

## Results

We analyzed 38 patients (17 men and 21 women) who were diagnosed with BRVO. Their ages averaged 67.2 ± 10.4 years (mean ± standard deviation). Twenty-eight age-matched healthy control subjects (14 men and 14 women) were included in the study. The baseline characteristics of patients with BRVO and healthy control subjects are shown in Table 1. The mean duration of disease was 2.7 ± 2.8 months, and 27 patients had superior BRVO and 11 had inferior BRVO.

**Table 1:** Clinical characteristics of patients with branch retinal vein occlusion and health subjects.

	BRVO	Healthy subjects
Number of eyes	38	28
Age (years)	67.2 ± 10.4	63.6 ± 6.2
Gender (men/women)	17/21	14/14
Duration of BRVO (months)	2.7 ± 2.8	
Best-corrected visual acuity (logMAR)	0.39 ± 0.30	
Location of BRVO (superior/inferior)	27/11	

Values are presented as the mean ± standard deviation.

*logMAR* logarithm of the minimum angle of resolution, *BRVO* branch retinal vein occlusion.

**Figure 1:**
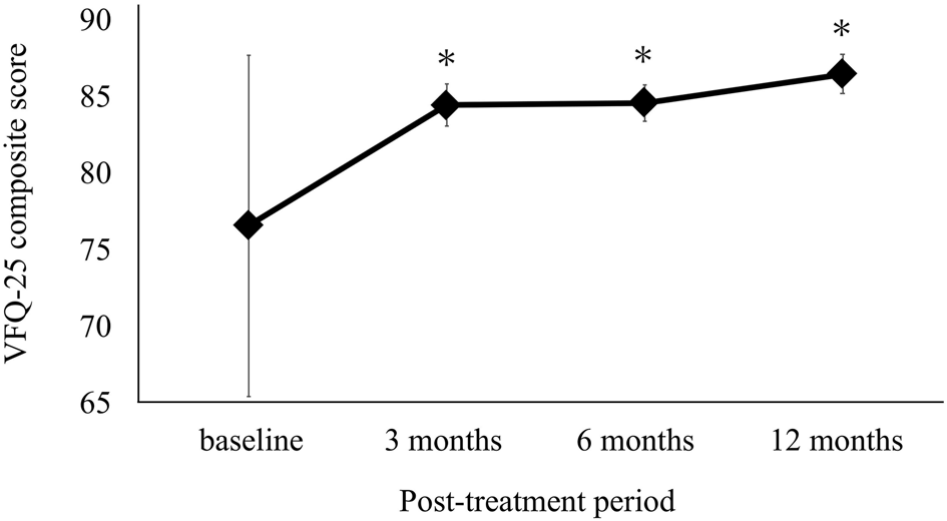
Time course of changes in the 25-item National Eye Institute Visual Function Questionnaire (VFQ-25) composite score in patients with branch retinal vein occlusion (BRVO). The VFQ-25 composite score improved significantly from 3 months after treatment (*P < 0.05; One-way repeated analysis of variance with Dunnett’s test).

**Figure 2:**
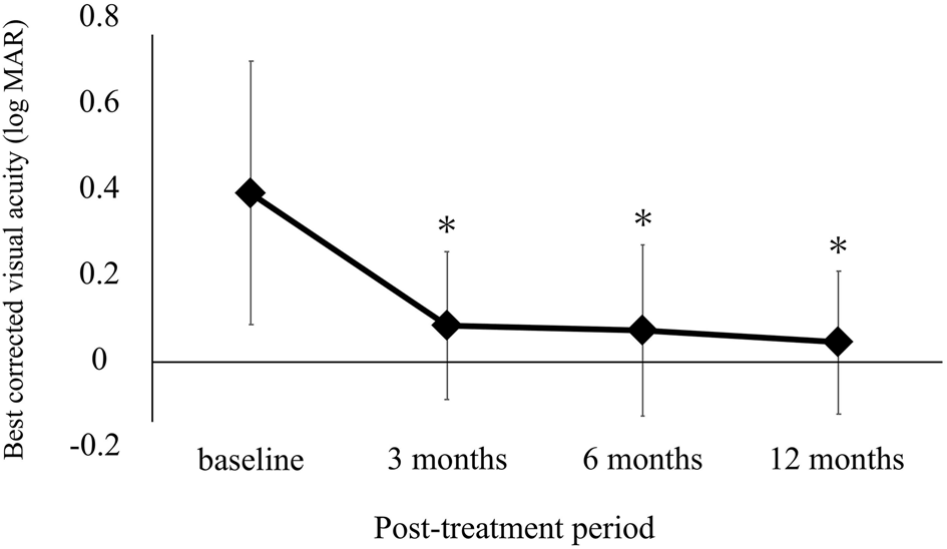
Time course of changes in best-corrected visual acuity (BCVA) in patients with BRVO. BCVA improved significantly 3 months after treatment (*P < 0.05; One-way repeated analysis of variance with Dunnett’s test).

**Figure 3:**
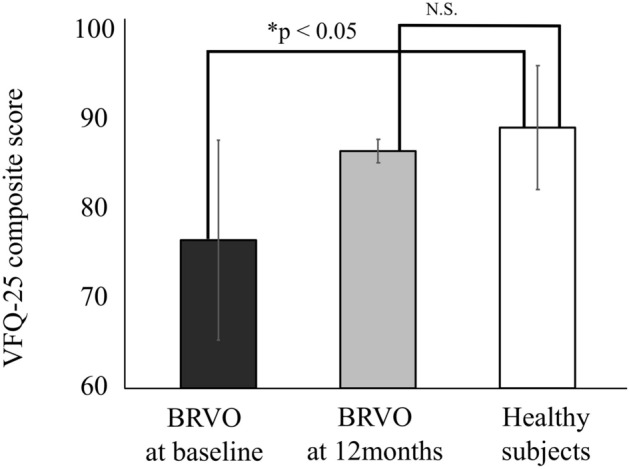
The VFQ-25 composite score in patients with BRVO before and after treatment and in healthy control subjects. *Mann–Whitney *U* test, *N.S.* not significant.

The time course of changes in the VFQ-25 composite score and BCVA in BRVO are shown in Figs. 1 and 2. IVR injections significantly improved VFQ-25 composite score from baseline (84.4 ± 8.4 at 3 months, 84.5 ± 7.4 at 6 months, 86.4 ± 7.8 at 12 months, *P* < 0.05) (Fig. 1). BCVA (Fig. 2) improved during the follow-up period from the baseline (*P* < 0.05). Figure 3 shows the VFQ-25 composite score for patients with BRVO at baseline and 12 months after treatment and for healthy subjects. The VFQ-25 composite score for patients with BRVO after treatment recovered to the level of healthy subjects (*P* = 0.20).

Table 2 shows the VFQ subscales for patients with BRVO at baseline and at 12 months and for healthy subjects. IVR significantly improved all VFQ subscale scores except for “general health”. There was no difference in VFQ subscales including “general health”, “general vision”, “ocular pain”, “distance vision”, “social functioning”, “role difficulties”, “dependency”, “driving”, “color vision”, and “peripheral vision” between patients with BRVO and healthy subjects. However, VFQ subscale scores of near vision and mental health were lower for patients with BRVO after treatment than for healthy subjects (*P* < 0.05, *P* < 0.01, respectively).

**Table 2:** The National Eye Institute 25-item visual function questionnaire subscales in healthy subjects and in patients with branch retinal vein occlusion at baseline and at 12 months after treatment.

VFQ-25 subscales	Healthy subjects	BRVO at baseline		BRVO at 12 months after treatment		
			P values as compared with healthy subjects		P values as compared with BRVO at baseline	P values as compared with healthy subjects
General health	62.5 (19.8)	50.0 (19.3)	< 0.05*	53.3 (14.4)	0.20	0.053
General vision	77.9 (9.9)	58.4 (17.9)	< 0.001*	74.6 (10.2)	< 0.001^†^	0.12
Ocular pain	83.5 (15.2)	78.9 (20.4)	0.59	85.5 (15.0)	< 0.05^†^	0.49
Near vision	87.5 (11.9)	64.4 (16.8)	< 0.001*	80.7 (13.4)	< 0.001^†^	< 0.05*
Distance vision	86.3 (12.0)	75.9 (11.1)	< 0.001*	86.4 (10.9)	< 0.001^†^	0.78
Social functioning	94.9 (8.3)	83.6 (16.0)	< 0.01*	88.9 (17.2)	< 0.05^†^	0.094
Mental health	93.8 (9.3)	71.9 (21.9)	< 0.001*	86.0 (12.2)	< 0.001^†^	< 0.01*
Role difficulties	91.1 (11.7)	81.4 (19.9)	0.096	92.2 (11.9)	< 0.0^†^	0.51
Dependency	96.7 (10.0)	86.7 (16.4)	< 0.01*	93.6 (9.6)	< 0.01^†^	0.055
Driving	86.0 (11.6)	77.4 (16.1)	0.091	87.2 (9.3)	< 0.005^†^	0.67
Color vision	95.6 (9.8)	88.2 (13.9)	< 0.05*	94.1 (10.1)	< 0.05^†^	0.53
Peripheral vision	86.6 (18.6)	75.0 (16.4)	< 0.01*	81.6 (12.9)	< 0.005^†^	0.11

Scores presented as mean (standard deviation).

*VFQ-25* 25-Item Visual Function Questionnaire.

*Significantly different from the healthy subjects (Mann–Whitney *U* test).

^†^Significantly different between BRVO at baseline and at 12 months after treatment (Wilcoxon signed-rank test).

**Figure 4:**
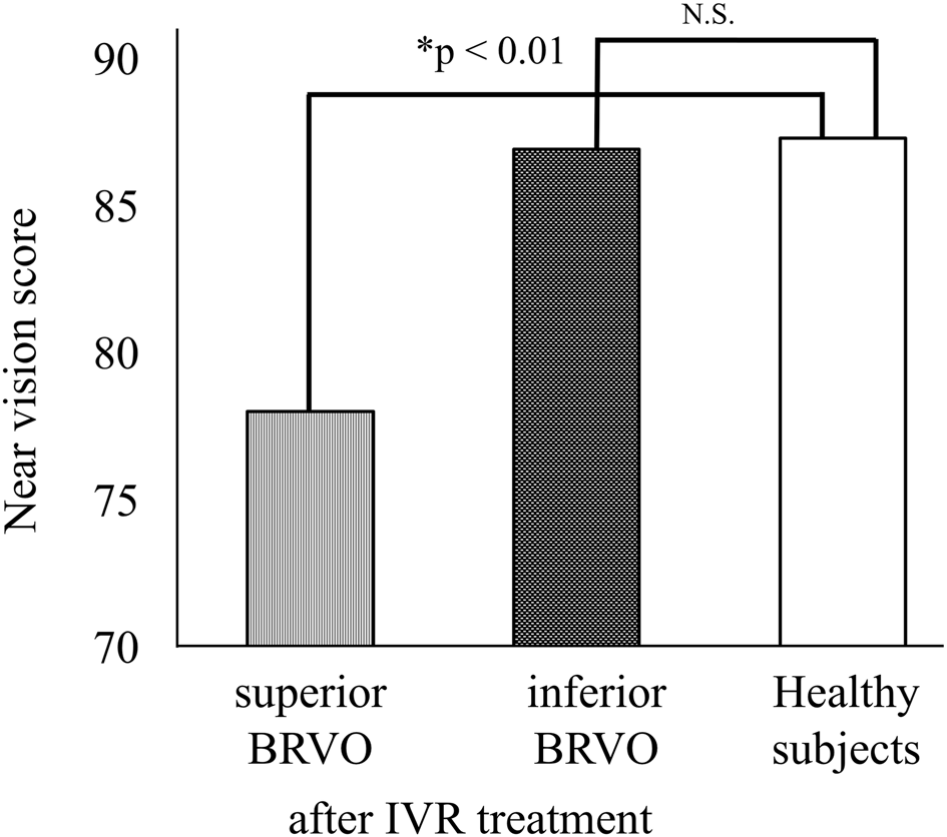
The near vision score in patients with superior and inferior BRVO after treatment and in healthy control subjects. *Mann–Whitney *U* test, *N.S.* not significant.

Patients who had superior BRVO (27 patients, 11 men, and 16 women, aged 67.0 ± 10.1 years) had 78.1 ± 12.5 points of “near vision”, and patients who had inferior hemorrhage (11 patients, 6 men, and 5 women, aged 67.6 ± 11.8 years) had 87.1 ± 14.1 points after 12 months. Figure 4 shows that there was a significant difference between the patients with superior BRVO and the healthy group in their “near vision” scores even after IVR treatment (*P* < 0.01). There was no relationship between the VFQ-25 composite score and BCVA in the treated eyes of patients with BRVO before and after treatment. The BCVA in fellow eye was 0.08 ± 0.42 and there was no relationship between the VFQ-25 composite score and BCVA in fellow eyes of patients with BRVO before and after treatment. The number of IVR injections was not correlated with the VFQ scores.

## Discussion

The mean VFQ-25 composite score was 76.5 ± 11.1 points (range 43.9 to 95.5) for patients with naïve BRVO at baseline in this study. Previous studies have reported that the mean VFQ-25 composite scores at baseline for patients with ERM, MH, DME, and PDR were 66.2, 70.5, 63.1, and 56.3, respectively^7–9,11^. Thus, the mean VFQ-25 composite score in BRVO was found to be relatively higher than in other retinal disorders.

Treatment of BRVO with IVR improved the VFQ-25 composite score and all subscale scores except for “general health”. An improvement from baseline in the mean VFQ-25 composite score was observed as early as 12 months, which accounted for 9.9 points in this study. Previous studies have reported that the mean change from baseline VFQ-25 composite score in patients with BRVO was 9.3 points and 10.4 points in the 0.3 mg and 0.5 mg ranibizumab treatment groups, respectively, in the BRAVO study^13^, while it was 9.4 points with aflibercept treatment in the VIBRANT study^16^. While our results were equally effective in determining the VR-QOL, there was a difference in regimens between our study and these two studies. The treatment period was day 0 to month 2 in this study, while it was day 0 to month 5 in the BRAVO study^13^ and day 0 to month 6 in the VIBRANT study^16^. Therefore, from the viewpoint of VR-QOL, IVR treatment with 3+ PRN may be adequate to treat patients with naïve BRVO.

The mean VFQ-25 composite score at baseline in BRVO was worse than that in the healthy subjects in this study. Okamoto et al.^7,11^ reported that the preoperative VFQ-25 composite scores were significantly lower in patients with ERM and PDR than in healthy subjects. Patients with BRVO after treatment had a good mean VFQ-25 composite score, similar to healthy subjects. A previous study had revealed that the VFQ-25 composite score remained significantly lower for the patients with ERM, MH, DME, RD, and PDR than the healthy subjects, even after surgery^18^. Therefore, unlike other retinal diseases, the treatment of BRVO with IVR can be considered to be favorable in terms of QOL prognosis.

As shown in the results, IVR treatment significantly improved almost all VFQ-25 subscale scores for patients with BRVO, similar to those in healthy subjects. However, only subscales “near vision” and “mental health” did not improve to normal level, even after treatment. Although the score of “near vision” of patients with inferior BRVO was the same as that of healthy subjects, that of patients with superior BRVO was lower than that of healthy subjects. Patients with superior BRVO had inferior visual field impairments. It is well known that humans use an inferior visual field for near work, such as reading books and detailed work^19,20^. Therefore, QOL for patients with superior BRVO with inferior visual field impairment may have been lower than that of healthy subjects.

In this study, there was no significant association between the VFQ-25 composite score and the BCVA of patients with BRVO before and after treatment. Frick et al.^21^ reported that VR-QOL score correlated positively with BCVA in patients with uveitis. Matza et al.^22^ revealed that changes in BCVA were associated with corresponding changes in VR-QOL among patients with diabetic retinopathy. Previous studies have revealed that contrast sensitivity affected VR-QOL of patients with DME and RD^9,10^. In addition, it is known that metamorphopsia is significantly associated with VR-QOL in ERM and MH^7,8^. Based on the results of these reports, visual functions such as metamorphopsia and contrast sensitivity might be associated with the VR-QOL in patients with BRVO; further studies are required to identify the visual function associated with VR-QOL.

One of the limitations of our study was the small sample size, which reduced the probability of identifying statistically significant associations. The placebo effect with the VFQ-25 can be a limitation of this study. The patients recognized that they had received IVR treatment and may have answered VFQ-25 questions more positively because of the expectation that they would benefit from the treatment. This could not be avoided by the study design and could have accounted for some of the improvements in the VFQ-25.

In summary, the VR-QOL was compromised in patients with naïve BRVO. Treatment with IVR improved the VFQ-25 composite score and subscales, except for “general health”, to normal levels. Visual acuity was not associated with the VR-QOL in patients with BRVO.

## Methods

This prospective study was approved by the Institutional Review Board of the University of Tsukuba Hospital and Mito Kyodo General Hospital and was performed in accordance with the Declaration of Helsinki. This study included patients who had been diagnosed with BRVO at the University of Tsukuba Hospital and Mito Kyodo General Hospital between December 2016 and December 2019. All patients were treatment-naïve and had no history of treatment. Exclusion criteria were as follows: (1) previous history of vitreoretinal surgery, (2) previous history of ophthalmic disorders except mild refractive errors and mild cataract, (3) history of macular edema treatment within 90 days prior to the commencement of the study (including sub-tenon triamcinolone acetonide, intravitreal bevacizumab, IVR, intravitreal aflibercept, topical steroid, carbonic anhydrase inhibitors), (4) history of intraocular surgery within 90 days prior to the commencement of the study, (5) contralateral eye with retinal vein occlusion (RVO), (6) poor control of hypertension and diabetes mellitus, and (7) history of laser treatment within 30 days prior to the commencement of the study. We also included age-matched healthy control subjects. Informed consent was obtained from all individual participants included in the study.

Elderly patients with a diagnosis of BRVO were examined every month and treated with IVR injection pro re nata (PRN) for 12 months. During the treatment period (day 0 to month 2), the patients received monthly IVR injections. During the observation period (3–12 months), the patients received monthly IVR if the central retinal thickness (CRT) was > 300 μm as measured by optical coherence tomography (OCT), or if the patient exhibited serous RD or subretinal hemorrhage. IVR was performed by two ophthalmologists (SM and TM).

The patients answered the VFQ-25 before treatment and at 3, 6, and 12 months after treatment. The research staff explained the questionnaire to the patients, gave verbal instructions, and provided assistance as needed. The completed questionnaires were checked by the research staff for any missing data. The VFQ-25 consists of 25 items where patients are expected to assess the level of difficulty due to specific visual symptoms and in daily activities. Each item is applied to one of the 12 subscales, namely, “general health”, “general vision”, “ocular pain”, “near vision”, “distance vision”, “social functioning”, “mental health”, “role difficulties”, “dependency”, “driving”, “color vision”, and “peripheral vision”. These subscales range from 0 to 100 points, with 100 indicating the highest function. The VFQ-25 composite score is the average score for all subscales, excluding questions regarding “general health”. In this study, a Japanese version of the VFQ-25 was used, which was adjusted for Japanese lifestyle and culture^6^.

We examined the best-corrected visual acuity (BCVA) at baseline and monthly, post-treatment. The retinal microstructure was measured using spectral domain OCT (Cirrus; Carl Zeiss, Dublin, CA), and five-line raster cross scans and macular cube were performed using Cirrus analysis software version 3.0, with more than 7/10 signal strength. Based on the OCT images, we measured CRT in a circle with a diameter of 1 mm. It was assessed by two special retinal ophthalmologists (TM and SM). The BCVA was measured with the Landolt chart and converted to the logarithm of the minimum angle of resolution (logMAR) units for analysis.

The mean and standard deviation were calculated for age, VFQ-25 composite score, 12 subscales, and the BCVA. The results of the VFQ-25 composite score were subjected to repeated measures analysis of variance to assess the course of the changes. If a significant difference was found, Dunnett’s test was performed. The Mann–Whitney *U* test was used to compare the VFQ-25 composite score between healthy subjects and patients with BRVO both at baseline and 12 months after IVR. The same test was used to compare all subscales between patients with BRVO and those with healthy subjects. The same test was used to compare the near vision scores of the VFQ-25 subscale between patients with superior and inferior BRVO and healthy subjects. The Wilcoxon signed-rank test was used to examine the differences in each subscale between baseline and after treatment. The association between the VFQ-25 composite score and BCVA in eyes with BRVO and that in fellow eyes was analyzed using Spearman’s rank correlation coefficient test. The same test was used to assess the relationship between the VFQ-25 composite score and the number of injections.

## Data Availability

The datasets generated during and/or analysed during the current study are no publicly available. If someone want to request the data from this study, please contact us at s0711715@yahoo.co.jp.
